# Investigation of Cylindrical Piezoelectric and Specific Multi-Channel Circular MEMS-Transducer Array Resonator of Ultrasonic Ablation

**DOI:** 10.3390/mi12040371

**Published:** 2021-03-30

**Authors:** Jian-Chiun Liou, Chih-Wei Peng, Zhen-Xi Chen

**Affiliations:** Department of Biomedical Engineering, School of Biomedical Engineering, Taipei Medical University, Taipei 11031, Taiwan; cwpeng@tmu.edu.tw (C.-W.P.); hoppyshi@gmail.com (Z.-X.C.)

**Keywords:** resonance, ablation, ultrasound MEMS-transducer

## Abstract

Background: A cylindrical piezoelectric element and a specific multi-channel circular microelectromechanical systems (MEMS)-transducer array of ultrasonic system were used for ultrasonic energy generation and ablation. A relatively long time is required for the heat to be conducted to the target position. Ultrasound thermal therapy has great potential for treating deep hyperplastic tissues and tumors, such as breast cancer and liver tumors. Methods: Ultrasound ablation technology produces thermal energy by heating the surface of a target, and the heat gradually penetrates to the target’s interior. Beamforming was performed to observe energy distribution. A resonance method was used to generate ablation energy for verification. Energy was generated according to the coordinates of geometric graph positions to reach the ablation temperature. Results: The mean resonance frequency of Channels 1–8 was 2.5 MHz, and the cylindrical piezoelectric ultrasonic element of Channel A was 4.2546 Ω at 5.7946 MHz. High-intensity ultrasound has gradually been applied in clinical treatment. Widely adopted, ultrasonic hyperthermia involves the use of high-intensity ultrasound to heat tissues at 42–45 °C for 30–60 min. Conclusion: In the ultrasonic energy method, when the target position reaches a temperature that significantly reduces the cell viability (46.9 °C), protein surface modification occurs on the surface of the target.

## 1. Introduction

For the treatment of deep hyperplastic tissues and tumors (e.g., breast cancer and liver tumors), ultrasound thermal therapy is a highly precise, highly accurate cardiac catheterization and treatment. The basic procedure of the fully developed radiofrequency treatment involves puncturing the peripheral artery of the hand or leg (usually the artery of the wrist or groin) under local anesthesia, and then inserting a tailored slender catheter into the blood stream along a large blood vessel to examine and treat it [1,2]. The catheter allows specially made equipment such as balloons and stents to enter the body and reopen blocked blood vessels (e.g., through balloon dilatation or stenting). Because of the characteristics of blood vessels, the rebound effect or crack formation may occur, which would likely entail relocking within a short time. Stent placement offers vascular stability to fully overcome the rebound problem, but endothelial cell growth or thrombus proliferation can lead to in-stent restenosis, which remains the toughest problem in cardiac catheterization. In response, the application of drug-eluting stents can considerably reduce in-stent restenosis from over 20% to less than 3%.

Limitations still exist in cardiac catheterization. For example, atherosclerosis or plaque accumulation can appear uniform in some patients, which renders examining the actual condition in blood vessels and the actual vessel diameter through cardiac catheterization imaging difficult, and may even affect the choice of intravascular stents. Intravascular ultrasound involves using a heart catheter with a miniaturized ultrasound probe attached to enter the deep veins for examination. Simultaneously displaying the vascular stenosis diagnosis (X-ray image) and intravascular tissue structure (soft plaque) on screen in the examination room facilitates confirmation of the scope, length, and wall size of the vascular stenosis [3,4,5,6]. This also enables physicians to select the appropriate treatment strategy before surgery (e.g., stent size) as well as performing postoperative checks for insufficient dilatation and incomplete lesion coverage, thereby reducing the incidence of vascular restenosis. Intravascular ultrasound is generally recommended for patients with complex conditions. According to overseas statistics, proper use of intravascular ultrasound can reduce the restenosis rate by approximately 10–30%, and thus the use of such technology is increasing. In terms of automatic picture transmission and three-dimensional (3D) reconstruction, 3D rotational angiography images are automatically transmitted to the workstation for image reconstruction to rapidly display 3D blood vessel images [7,8,9,10]. This new technology may serve as a clinical evaluation and treatment method before and after surgery and will soon become a trend in X-ray-dependent cardiac catheterization.

Among the various thermal sources that can be used for ablation of the hyperplastic tissue between the left atrium and the pulmonary vein, ultrasound has particularly high potential for development. Ultrasound is sound waves at frequencies of 20,000 Hz or higher, which are inaudible to the human ear. Therapeutic ultrasound can produce two types of biological effects, namely thermal and nonthermal effects. Lead zirconate titanate (PZT) ceramics for ultrasonic-induced nonthermal effects can be further classified into cavitation, acoustic streaming, acoustic torque, radiation force, and radiation pressure [11,12,13,14,15,16,17,18,19]. These nonthermal phenomena are the result of attenuated ultrasound energy after ultrasound is absorbed by the biological tissues or scattering while traveling through biological tissues. Energy absorbed by the tissues is converted into thermal energy that increases the temperature of biological tissues, causing thermal effects [20,21,22,23,24,25,26,27,28].

## 2. Framework of the Ultrasound Ablation System

Ultrasound-induced thermal effects differ from the traditional thermal effect. The traditional thermal effect requires thermal energy from the surface of the target, and the heat gradually penetrates into its interior. A relatively long period of time is required for the heat to be conducted to the target area. When the target position reaches a temperature (42 °C) that significantly reduces cell viability, the surface of the target has already been overheated. Cauterization of tumors can damage normal cells. By contrast, the short wavelength of ultrasound enables energy to be focused on an extremely small area through phase adjustment, allowing the energy to be transferred effectively to the soft tissue. This prevents harm to normal tissues, through which the ultrasound passes. Therefore, for the treatment of deep-seated tumors (e.g., breast cancer and liver tumors), ultrasound thermal therapy is of optimal development potential [29,30,31,32,33,34,35,36,37,38,39,40].

The following paragraphs describe the principle, function, and effect of ultrasound introduction. In daily life, the terms “acoustic wave” and “sound wave” are often heard, but people generally have a limited understanding of them. Simply put, a sound wave is the frequency of a substance generated through mechanical vibration. Audible sound waves to human beings generally have vibration frequencies from 20 Hz to 18,000 Hz per second and are termed “subsonic waves”. Ultrasound refers to sound waves with a vibration frequency of 20,000 Hz or higher. In recent years, ultrasound applications have occupied a significant place in medical cosmetology because ultrasound-induced thermal and mechanical vibrations reach a permeability level beyond that of other instruments. Because ultrasound penetrates deep into tissues and transforms into thermal energy, it provides efficient, deep massages and maximizes drug efficacy [41].

### 2.1. Mechanism of Ultrasound Introduction

#### 2.1.1. Ultrasound Heating

During the propagation of ultrasound, drugs, media, skin, and subcutaneous tissues absorb ultrasound energy and convert it into thermal energy, which increases the kinetic energy of drugs and carbohydrates, lipids, and proteins in skin cell membranes. Increased skin temperature enlarges pores and diameter of the sweat gland ducts, thereby accelerating the diffusion of drugs through percutaneous absorption. The thermal effect produced activates deep-tissue cells, strengthens local metabolism, and accelerates the absorption of drugs [42].

#### 2.1.2. Mechanical Effect

Ultrasound can temporarily change the interstitial lipid structure of the skin’s keratinocytes, increase the effective penetration area, increase the permeability of cell membranes, enlarge the tiny pores between epidermal cells, and accelerate the absorption of skin care products. Ultrasound can cause high-speed oscillation of drugs, media, and intracellular particles; reduce the electric potential and increase the permeability of cell membranes; and facilitate percutaneous absorption of skin essences [43].

#### 2.1.3. Convective Transport or Streaming

Under the effect of ultrasound, bubbles in the diffusion system vibrate continuously, causing particle rotation and liquid circulation around the bubbles, which facilitate the convective transport of drugs into the skin through diffusion. In addition, high-frequency physical vibrations produce slight massage effects and promote blood and lymphatic circulation to achieve repair effects [44].

#### 2.1.4. Cavitation Effect

The cavitation effect refers to the ultrasound-induced vibration of gas molecules and bubbles in media and cells, which causes the bubbles to rupture and form voids or air sacs. This effect changes the lipid structure of the skin, causing the formation of media cavitation outside the skin. Numerous water molecules enter the lipid structure to form water-soluble channels, which facilitate the percutaneous penetration of drugs. Furthermore, the effective diffusion area of skin is greatly enlarged, which facilitates the percutaneous absorption of skin essences [45].

## 3. Experiment and Results

Microelectromechanical systems (MEMS)-transducer-driven multiplex bioelectronics signals ultrasonic ablation systems integrated with precision medicine can be divided into (1) a cylindrical piezoelectric ultrasonic element (Channel A) and (2) a 16-channel circular MEMS-transducer array. Thermal energy was generated by accumulating ultrasound energy at the front end of the catheter. Using novel ultrasonic cardiac microcatheters, ultrasound ablation was performed to measure the protein modification.

In clinical application, (1) when the patient’s symptoms are severe and the area to be treated is large, a cylindrical piezoelectric ultrasonic element will be used. An entire cylinder performs ablation. (2) When the patient’s symptoms are mild and the treatment area is small, a 16-channel circular MEMS-transducer array will be used. Because there is only a very small area in the blood vessel to be ablated. When using 16-channel circular MEMS-transducer array, piezoelectric systems are designed to drive signals to operate individual channels.

In this study, these two forms of piezoelectric ultrasonic system were employed for ultrasonic energy generation and ablation. In the first stage, beamforming was performed to observe energy distribution; then, in the second stage, a resonance method was used to generate ablation energy for verification. Energy was generated according to the coordinates of geometric graph positions to reach the ablation temperature.

Sensor arrays for directional signal transmission or reception constitute a beamforming detection system. The technology involves adjusting the parameters of the basic phased array units while energy from other angles suffers destructive interference. Beamforming can be used on the transmitter and receiver.

Regarding the supply voltage and resonance frequency of the cylindrical piezoelectric ultrasonic element (Channel A), the loop impedance is 4.2546 Ω and the resonance frequency is 5.7946 MHz. For the 16-channel circular MEMS-transducer array, the resonance frequency characteristics and loop impedance characteristics of each channel were measured first, and bioelectronics signals corresponding frequencies were delivered to the piezoelectric elements. The minimum loop impedance structure enabled resonance signals to be measured for monitoring resonance energy.

### 3.1. Channel A Circular MEMS-Transducer

**Figure 1:**
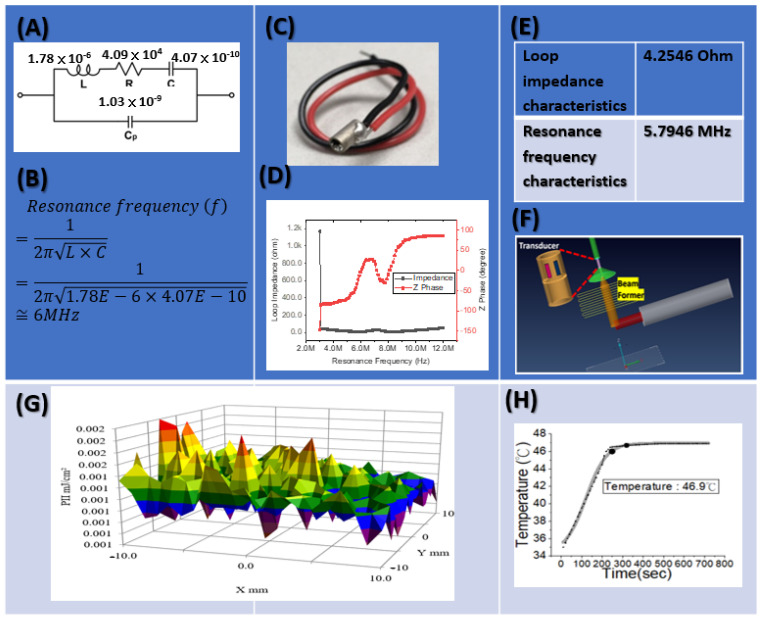
Single-channel cylindrical piezoelectric ultrasonic element (Channel A). (**A**) Measure resonance frequency model, The Butterworth-Van Dyke (BVD) equivalent circuit extracted data of the actual elements and calculated the equivalent resistor, inductor, and capacitor (RLC) elements. The advantage of the BVD equivalent circuit is that the circuit can be analyzed quickly. (**B**) Parameters, Cp: capacitance between the two electrode plates of the piezoelectric element, R: impedance is equal to R at resonance, C: capacitance is equal to C at resonance, L: inductance is equal to L at resonance. (**C**) Cylindrical piezoelectric ultrasonic element, (**D**) Loop impedance of Channel A, (**E**) Resonance condition, (**F**) Beam former scanning, (**G**) Energy profile, (**H**) Temperature observed.

The supply voltage and resonance frequency conditions of Channel A are depicted in Figure 1. Specifically, the loop impedance and resonance frequency are 4.2546 Ω and 5.7946 MHz, respectively.

The output pulse bioelectronics signals of an application-specific integrated circuit (ASIC) have a resonance frequency of 6 MHz and supply voltage of 108.0 V. Thermal energy was generated by accumulating ultrasound energy at the front end of the catheter. Using ultrasonic cardiac microcatheters, ultrasound ablation was performed to measure the protein modification. The temperature control result and corresponding sound intensity are presented in Figure 1, which shows that the temperature reached 46 °C at heating durations of 180 s, 220 s, and 240 s. According to the results, temperature overshoot was observed at 200 s. Because of the short heating duration, the output intensity was progressively incremental, and a strong output intensity per second was observed before the target temperature was reached. When the target temperature was reached, the output intensity dropped sharply but not fast enough, causing temperature overshoot.

The parameter settings were as follows: a focused ultrasound frequency of 6 MHz; a fixed heating duration of 220 s; and target temperatures of 45 °C, 55 °C, and 60 °C. The temperature control results are presented in Figure 1, demonstrating that the controller could reach the target temperature of 46 °C or even higher within 220 s. Changes in an ablation experiment conducted on a pork specimen under different combinations of parameter settings were recorded. The diameter and thickness of the lesion were measured through sonography and actual measurement with a ruler.

The diameter and thickness of the lesion yielded from the ultrasound image were compared with the actual measurement to determine their correlation.

The preliminary experiment results of ultrasound catheter ablation were as follows. At 46 °C (1/4 damage) or 50 °C (10/60 damage), the pulse was terminated prematurely because of catheter heating (thermocouple). The output pulse bioelectronics signals of an application-specific integrated circuit (ASIC) have a resonance frequency of 5.7946 MHz and supply voltage of 108.0 V. For the single-channel cylindrical piezoelectric ultrasonic element (Channel A), the ultrasonic frequency is 5.7946 MHz, and the transmission speed is 2 kHz. That is, 500 us sends out a pulse high voltage of 5.7946 MHz. The launch speed of pulse is 500 us once. The pulse width of the application-specific integrated circuit (ASIC) drive system determines the frequency of the ultrasound. The width of each pulse is (1/5.7946 MHz)/2. The pulse width of each pulse signal is about 172 ns. The diameter of the damage caused by temperatures of 60–70 °C for 180–240 s was approximately 3 cm, but poor reproducibility was observed at 60 °C. The depth of the lesion was determined by the position of the crystal. Considering that the crystal angle caused ultrasonic beam tilt, an eccentric crystal position may have affected the temperature feedback output by the thermocouple because of temperature unevenness. Consequently, the size of the lesion was reduced. Single-channel cylindrical piezoelectric ultrasonic element (Channel A) is shown in Figure 1.

### 3.2. 16-Channel Circular MEMS-Transducer Array

**Figure 2:**
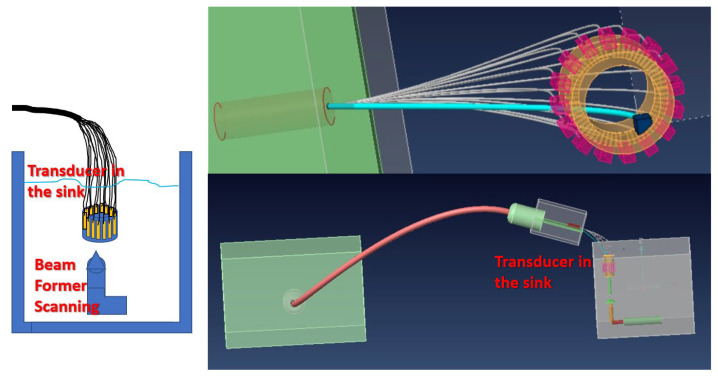
Specific 16-channel circular microelectromechanical systems (MEMS)-transducer array.

First, the resonance frequency characteristics and loop impedance characteristics were measured, and the bioelectronics signals corresponding resonance frequencies were delivered to the piezoelectric elements. The minimum loop impedance structure enabled resonance signals to be measured for monitoring resonance energy. The specific 16-channel circular micro-MEMS-transducer array is shown in Figure 2.

**Figure 3:**
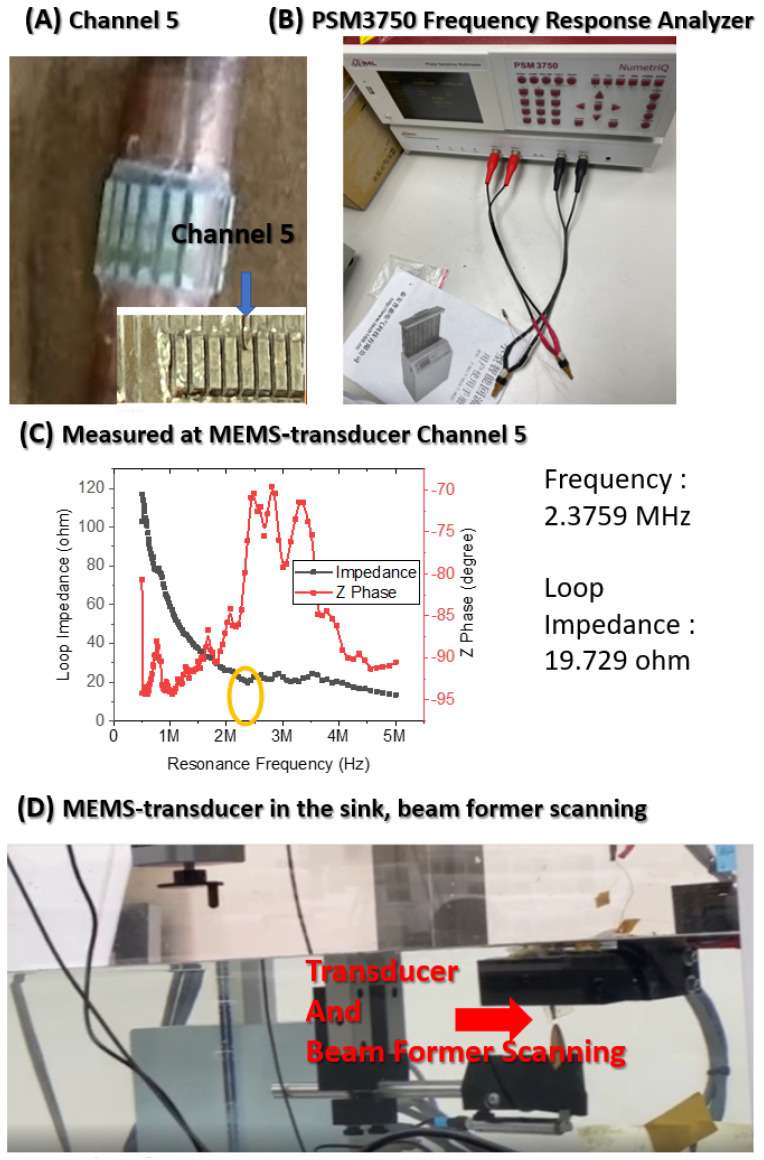
Channel 5 generated by ultrasonic energy was selected to increase the voltage supply. (**A**) The 16-channel circular MEMS-transducer array; (**B**) The PSM3750 Frequency Response Analyzer (Miko-Kings Instruments LTD, Fotan, Shatin, N.T, Hong Kong) was used for measuring the resonance frequency of the piezoelectric element; (**C**) Measured at MEMS-transducer Channel 5, the frequency and impedance at the resonance frequency were 2.3759 MHz and 19.729 Ω, respectively; (**D**) After putting the MEMS-transducer in the sink, beamforming was performed to scan its ultrasonic resonance energy.

This study chose to increase the voltage supply to Channel 5 as shown in Figure 3 (A), which generated satisfying ultrasonic energy in the first experimental stage, to drive the piezoelectric element, which had a resonance frequency and loop resistance of 2.3759 MHz and 19.729 Ω, respectively (Figure 3).

**Figure 4:**
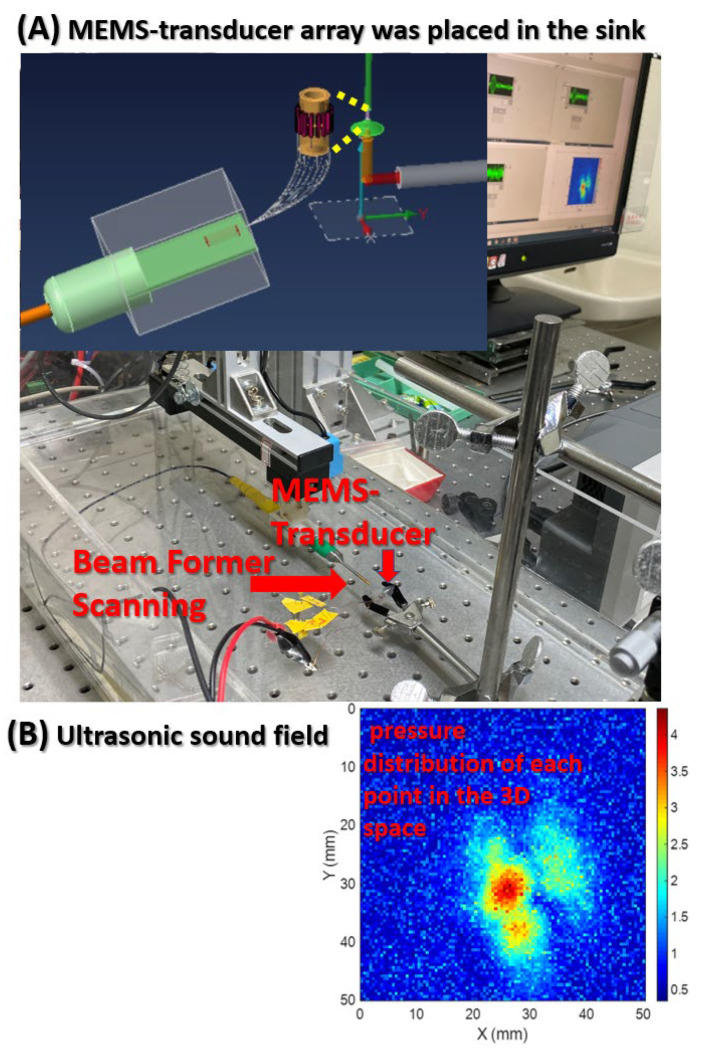
Beamforming was performed to scan the ultrasonic resonance energy of the MEMS-transducer. (**A**) The 16-channel circular MEMS-transducer array was placed in the sink for ultrasonic signal monitoring. (**B**) 10.00 Vpp signals were sent to Channel 5 at a frequency of 2.3759 MHz. Beamforming was performed to detect ultrasound signals.

Beamforming was performed to scan the ultrasonic resonance energy of the MEMS-transducer and to detect ultrasound signals, as shown in Figure 4.

This figure presents the framework of the ultrasonic sound field scanning system. This study used graphical programming language in LabVIEW; connected a needle hydrophone to the XYZ three-axis platform to measure the pressure distribution of each point in the 3D space; and built a system for the automatic measurement of ultrasonic sound fields. Beamforming can be interpreted as a technique based on wave interferences, which achieve the optimal performance on the receiving end through phase adjustment. The geometric interpretation of singular value decomposition (SVD) is that SVD can be performed to switch any two perpendicular coordinates within a matrix (M) to another two perpendicular coordinates. Changing the coordinate axis of matrix A (m × n), using P as the transformation matrix, an m × n-dimensional space can be transformed into another m × n-dimensional space, thereby creating similar changes in spatial rotation or skewness within the space.

## 4. Discussion

In this study, the PSM3750 Frequency Response Analyzer was adopted to measure the resonance frequency of the piezoelectric element. Subsequently, the obtained resonance frequency conditions were applied to provide high voltages (±20 V to ±50 V) and enable resonance frequency operations.

This study selected eight channels to measure the resonance frequency conditions and supply voltage, as shown in Table 1. It mainly analyzes the resonance frequency condition comparison between Channels 1–8 and Channel A. The measurement parameters include loop impedance characteristics, resonance frequency characteristics, each loop in a multi-channel architecture, and a single-channel loop.

**Figure 5:**
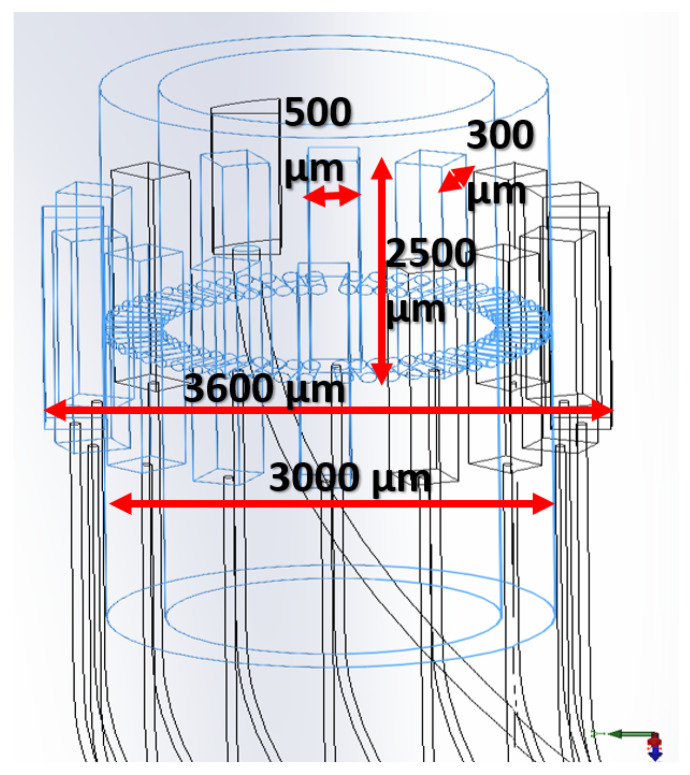
Geometry of MEMS-transducer array, 16 pcs lead zirconate titanate (PZT) arranged in a ring to form a tube.

**Figure 6:**
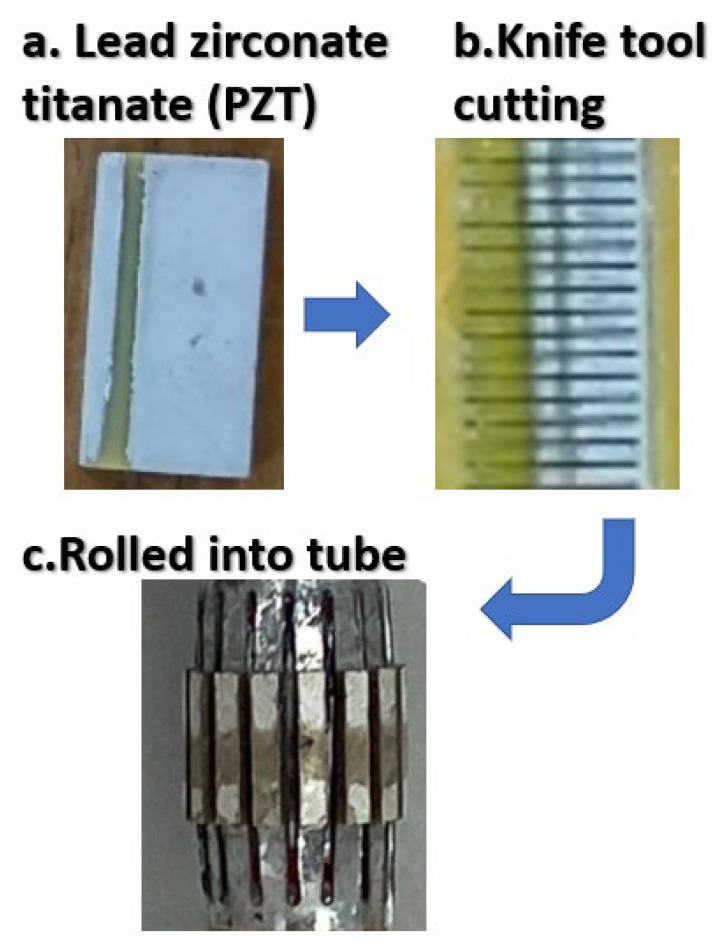
Fabrication steps. (**a**) Lead zirconate titanate (PZT); (**b**) Knife tool cutting; (**c**) Rolled into tube.

Table 1 presents two measured parameters. The values of these two parameters are the loop impedance characteristics and the resonance frequency of eight individual channels. From the technical analysis, the piezoelectric ceramic powder used in the ultrasonic bonding technology is mainly composed of lead zirconate titanate (PZT), and then lead oxide (PbO), zirconium oxide (ZrO2), and titanium oxide (TiO2) are added. After the powders are mixed, they are compressed into various specifications and shapes with a press, and sintered at a temperature of about 1350 °C. The resulting product is polarized into piezoelectric ceramics. However, piezoelectric materials are susceptible to energy released by electric energy, mechanics, and thermal effects, causing the resonance frequency of piezoelectric ceramics to drift. This study measured the resonance frequency between 2 MHz and 6 MHz. The corresponding impedance value of the entire loop is between 4 ohm and 500 ohm in these eight channels. The dimensions, geometry, materials, and fabrication details for the MEMS-transducer array are described below.

(A) Dimensions. The composition dimensions of the tube are as follows.
  Inner diameter: 3000 μm
  Outer circle diameter: 3600 μm
  The length of each pcs: 2500 μm
  Width: 500 μm
  Thickness: 300 μm
(B) Geometry. 16 pcs lead zirconate titanate (PZT) are arranged in a ring to form a tube, as shown in Figure 5.
(C) Materials. During the development process of piezoelectric materials, the new piezoelectric ceramic system is developed and prepared by low-temperature sintering. The manufacturing process reduces the sintering temperature of the material system by replacing elements inside the material or adding sintering aids. It is observed that the characteristics of the material change during low-temperature sintering. It explores the mechanism of characteristic changes and selects a low-temperature or high-temperature ceramic material system suitable for use in cardiac catheters. Piezoelectric ceramic powder is mainly composed of lead zirconate titanate (PZT). Its manufacturing process adds lead oxide (PbO), zirconium oxide (ZrO2), and titanium oxide (TiO2).
(D) Fabrication. The steps of precision machining are shown in Figure 6.
  - Lead zirconate titanate (PZT); the solid-state reaction method is used for preparation, the sintering needs to be performed in a high-temperature environment; for example, the sintering temperature of Pb(ZrTi)O3 is 1200 °C to 1350 °C.
  - Knife tool cutting; the machining shape to be produced by machining is determined by the tool shape. Because the ultrasonic processing is generated by the vibration of the tool indirectly through the grinding fluid to impact a shape. The machining shape and size will vary owing to the size of the abrasive, and the selection of the tool material is very important.
  - Rolled into tube; according to the application of treating deep hyperplastic tissues and tumors (such as breast cancer and liver tumors), the multi-channel piezoelectric sheet is rolled into a tube shape.
(E) In terms of materials and methods, the piezoelectric ceramic powder used in ultrasonic bonding technology is mainly composed of lead zirconate titanate (PZT), and then lead oxide (PbO), zirconium oxide (ZrO2), and titanium oxide (TiO2) are added. After mixing the powders, they are compressed into various specifications and shapes with a press, and sintered at a temperature of about 1350 °C. As the content of sintering aid Li2CO3 increases, it will help to improve the density of the test piece and related characteristics. This reflects that the sintering aid uses the mechanism of liquid phase assisted sintering to increase the sintering stability. Furthermore, the holes and voids produced by the test piece in the low temperature sintering environment are eliminated. As the content of Li2CO3 continues to increase, the electrical properties such as d33 and kp will decrease accordingly. This is because, if the doping of elements occupies the A site in the perovskite structure, such as common elements La^3+^, Cd^2+^, and Bi^2+^, they are called donor dopants. On the contrary, if it occupies the B site in the perovskite structure, such as common elements Fe^3+^, Ni^2+^, Zn^2+^, and Li^+^, they are called acceptor dopants. In this system, replacing the elements of B site2+ with lower valence Li+ will produce oxygen vacancies, which will limit the rotation of the domain. This causes the piezoelectricity value to decrease. When the amount of Li2CO3 added exceeds 0.6 wt%, the coercive electric field value will continue to rise, making the piezoelectric material “hard”. This is the so-called hard modification addition, and this micro-doping mechanism can be used to control the piezoelectric properties of different material systems, and the resulting product can be polarized into piezoelectric ceramics. Supplying a high frequency signal to operate this system, the resonant frequency of piezoelectric ceramics occurs.

**Figure 7:**
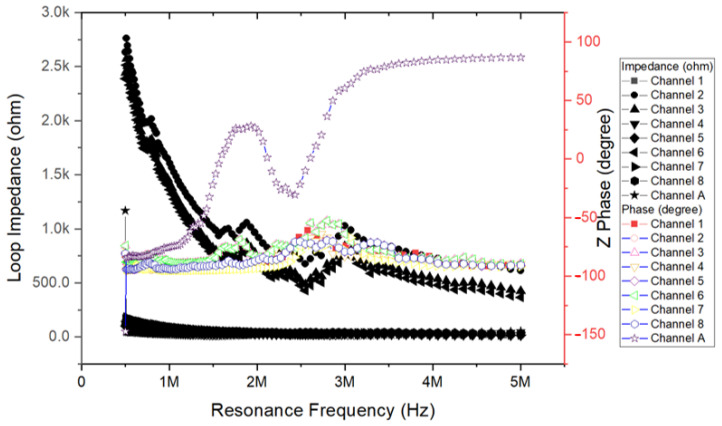
Impedance Z phase frequency conditions between Channels 1–8 and Channel A.

The chart of Figure 7 is a characteristic of the impedance and phase of near the resonance frequency. Cylindrical piezoelectric and specific multi-channel circular MEMS-transducer array resonator of ultrasonic is shown in Figure 1 to Figure 3. The measurement result curve distribution on the vertical axis is impedance and Z phase. The value of these curve distributions in research is to optimize the ultrasonic system when manufacturing MEMS transducers. These two parameters vary with the frequency. According to this result, the circuit channel of the piezoelectric element can be immediately fed with a signal waveform to generate a sound field.

The mean resonance frequency of Channels 1–8 was 2.5 MHz. This study proposed an innovative system focusing on the integration of precision medicine with multiplex bioelectronics signals ultrasonic ablation system driven by an array MEMS-transducer [46,47,48,49]. An ASIC-based cardiac microcatheter ultrasound ablation system was integrated with eight-channel ASIC, high-voltage and high-power output, design of piezoelectric devices, and design for testability to create a large array MEMS-transducer system. The output pulse signal of ASIC at a resonance frequency is 1/405.0 ns Hz and the supply voltage is 108.0 V. Thermal energy was generated by accumulating ultrasound energy at the front end of the catheter. Using ablation treatment based on ultrasonic cardiac microcatheters, this study performed ultrasound ablation-based protein modification measurements and actual catheter operation to explore the range of pixel ablation, which allowed the position and intensity of the ultrasonic pixels to be separately adjusted. Protein modification of the pork specimens was performed through ultrasonic ablation. The proposed innovative system was based on arrays of ultrasonic pixels for ablation, which enabled the individual adjustment of the position and intensity of the pixels. The range of pixel ablation surpassed a specific point or line and extended to the entire area.

The resonance frequency characteristics of the eight channels were required to conform to the frequency of the frequency generator, which required changes in vibration system characteristics and vibration modes, and thus affected the transfer of ultrasonic vibration energy. Ultrasound ablation is an extremely safe and minimally invasive treatment and involves precisely inserting ultrafine catheter electrodes into the pulmonary vein or superior vena cava area. During the treatment, the piezoelectric ceramic MEMS-transducer of the catheter emits ultrasound, and the tissues that ultrasound passes through generate heat from ion-induced turbulence. Consequently, the temperature in the target area begins to rise; when it reaches 60 °C or higher, the tissues therein (including hyperplastic tissues) are burned and become necrotic. Because the temperature is not excessively high, the risks of damaging adjacent tissues outside the target area (e.g., blood vessels) are low.

Tissue ablation is achieved by focusing high-energy ultrasound waves onto the target, allowing the tissue to absorb the energy and convert it into thermal energy, which increases the temperature in the focal area and causes coagulation necrosis of tissues. According to the system’s arrangement of array elements, ultrasound focus differs in treatment range and depth. Therefore, sound field measurement is necessary for the analysis of sound field distribution in ultrasound thermal therapy. High-intensity ultrasound has gradually been applied in clinical treatment. Widely adopted, ultrasonic hyperthermia involves the use of high-intensity ultrasound to heat tissues at 42–45 °C for 30–60 min. Many studies have explored high-intensity focused ultrasound (HIFU), which employs a large-area probe with superior geometric focusing that facilitates high energy emission and accuracy of lesion positioning [50]. Moreover, no cumulative dose damage occurs from multiple treatments, and ultrasound imaging can be performed simultaneously during treatment. In addition to destroying tumor tissues, HIFU has a superior hemostatic effect, which is effective in treating internal bleeding caused by the rupture of large blood vessels. Clinical applications of HIFU include treatment for benign or malignant tumors (e.g., liver cancer, pancreatic cancer, and sarcoma) as well as hemostasis, thrombolysis, and drug and gene delivery [51,52].

## 5. Conclusions

This study satisfied the supply voltage and resonance frequency requirements of a single-channel cylindrical piezoelectric ultrasonic element (Channel A). The loop impedance and resonance frequency of Channel A were 4.2546 Ω and 5.7946 MHz, respectively. For the 16-channel circular MEMS-transducer array, resonance frequency characteristics and loop impedance characteristics of each channel were measured first, and corresponding frequencies were delivered to the piezoelectric elements. The output pulse bioelectronics signals of an application-specific integrated circuit (ASIC) have a resonance frequency of 5.7946 MHz and supply voltage of 108.0 V. For the single-channel cylindrical piezoelectric ultrasonic element (Channel A), the ultrasonic frequency is 5.7946 MHz and the transmission speed is 2 kHz. That is, 500 us sends out a pulse high voltage of 5.7946 MHz. The launch speed of the pulse is 500 us once. The pulse width of the application-specific integrated circuit (ASIC) drive system determines the frequency of the ultrasound. The width of each pulse is (1/5.7946 MHz)/2. The pulse width of each pulse signal is about 172 ns. The minimum loop impedance structure enabled resonance signals to be measured for monitoring resonance energy. When the hyperplastic tissue reached a temperature of 42 °C, the cell viability tended to decline at first and then level off. This was because the heat tolerance of cells enabled cell tissue to gradually adapt to the environmental temperature of 42 °C. However, when the temperature rose to 46 °C, the cell viability decreased considerably and cells were no longer tolerant of heat. That is, cells cannot tolerate a temperature of 46 °C or above, which results in cell death. Therefore, heat can be utilized to treat hyperplastic tissues. In hyperthermia therapy for hyperplastic cells or tumors, when the mechanical energy of high-intensity ultrasound is converted into thermal energy in the medium, the high temperature generated by the ultrasonic energy prompts the target to rapidly solidify hyperplastic cell histones, forming irreversible tissue necrosis, thereby promoting cell proliferation and treating tumors.

## Figures and Tables

**Table 1:** Comparison of resonance frequency conditions between Channels 1–8 and Channel A.

	Channel 1	Channel 2	Channel 3	Channel 4	Channel 5	Channel 6	Channel 7	Channel 8	Channel A
Loop impedance characteristics	19.588 Ohm	675.14 Ohm	487.30 Ohm	23.744 Ohm	19.729 Ohm	429.05 Ohm	27.551 Ohm	39.376 Ohm	4.2546 Ohm
Resonance frequency characteristics	2.3759 MHz	2.8619 MHz	2.8619 MHz	2.5476 MHz	2.3759 MHz	2.5476 MHz	2.4890 MHz	2.3490 MHz	5.7946 MHz

## Data Availability

Not applicable.
